# Distorting temporal fine structure by phase shifting and its effects on speech intelligibility and neural phase locking

**DOI:** 10.1038/s41598-017-12975-3

**Published:** 2017-10-17

**Authors:** Yingyue Xu, Maxin Chen, Petrina LaFaire, Xiaodong Tan, Claus-Peter Richter

**Affiliations:** 1 Northwestern University, Department of Otolaryngology, 320 E. Superior Street, Searle 12-561, Chicago, IL 60611 USA; 2 Northwestern University, Department of Biomedical Engineering, 2145 Sheridan Road, Tech E310, Evanston, IL 60208 USA; 3 Northwestern University, The Hugh Knowles Center, Department of Communication Sciences and Disorders, 2240 Campus Drive, Evanston, IL 60208 USA

## Abstract

Envelope (E) and temporal fine structure (TFS) are important features of acoustic signals and their corresponding perceptual function has been investigated with various listening tasks. To further understand the underlying neural processing of TFS, experiments in humans and animals were conducted to demonstrate the effects of modifying the TFS in natural speech sentences on both speech recognition and neural coding. The TFS of natural speech sentences was modified by distorting the phase and maintaining the magnitude. Speech intelligibility was then tested for normal-hearing listeners using the intact and reconstructed sentences presented in quiet and against background noise. Sentences with modified TFS were then used to evoke neural activity in auditory neurons of the inferior colliculus in guinea pigs. Our study demonstrated that speech intelligibility in humans relied on the periodic cues of speech TFS in both quiet and noisy listening conditions. Furthermore, recordings of neural activity from the guinea pig inferior colliculus have shown that individual auditory neurons exhibit phase locking patterns to the periodic cues of speech TFS that disappear when reconstructed sounds do not show periodic patterns anymore. Thus, the periodic cues of TFS are essential for speech intelligibility and are encoded in auditory neurons by phase locking.

## Introduction

Speech is a robust signal that remains intelligible despite various means of perturbation^1^. Key components necessary for speech intelligibility have been examined. For example, speech containing only the envelope information (E, slow varying components < 50 Hz) or the temporal fine structure information (TFS, rapid varying components > 50 Hz) of a few frequency bands was found to be intelligible for normal hearing listeners^2–4^. The role of TFS has also been extensively examined through other hearing tasks. These tasks have shown that TFS is essential for speech intelligibility in noise, music perception, sound localization, and frequency discrimination. To demonstrate this, the low frequency spectrum (50–500 Hz) of TFS has been isolated and defined as conveying periodic cues by some researchers, also known as the temporal periodicity^5–10^. These periodic cues were specifically examined since they are abundant in human speech, music and even non-animated sounds due to physical constraints^7^. Findings from these experiments suggested that periodic cues are important for the perception of rhythm and syllabicity^5,11^, pitch, voicing, intonation, sound localization, and identifying a common sound source that activates different frequency bands under noise^6,7,12–15^.

The significance of TFS for speech intelligibility has been demonstrated through human behavior studies^8^. Studies have also been carried out to examine the underlying neural mechanisms related to TFS, specifically the periodic cues. Recent studies, which used Magneto- and electroencephalography showed clear representations of TFS in the recorded cortical activity^9,10^. Other studies attempted to assess the internal representation of the TFS using physiologically-plausible models of the auditory periphery^16–19^. More direct and fundamental neural correlates to the TFS cues have been explored in animals by measuring the discharge patterns of auditory neurons, in particular the timing of neural discharges or the phase locking of the neurons^20^. Phase locking is a phenomenon that is preserved along the auditory pathway from the cochlea to the cortex^21–29^. It is referred to as the tendency for auditory neurons to fire within a well-defined time window relative to a period of the stimulating frequency. This is particularly evident in the neural response to low-frequency pure tones^20^. Phase locking pattern is also seen in auditory nerve fibers in response to periodic sounds, such as modulated frequencies and steady-state vowels. Importantly, the TFS of natural speech sentences contains multiple representative frequencies, including the fundamental frequency (*F*_0_) and its harmonics. Auditory neurons may discharge at the rate of these frequencies^21,30–32^, i.e., phase lock to these frequencies. Thus, the phase locking pattern could serve as a tool in examining the neural coding of the TFS in speech.

While phase locking could be used to investigate the neural components of TFS, reconstructed speech sounds could be used to investigate its perceptual functions. Previous studies have frequently manipulated speech sounds to assess the perceptual role of different acoustic cues. Modified speech could be achieved by keeping the acoustic cues of interest, while disrupting or replacing other acoustic components, and vice versa. For example, studies examining E have reconstructed acoustic signals by replacing spectral cues with modulated sinusoids or pulse trains^33^ or spectrally matched noise^4^. The E is usually extracted by a Hilbert transformation or by the low pass filtering of the rectified bandpass filtered speech signal. Through dividing the E from the original signal or peak clipping, TFS cues could be isolated. An alternative method to distort speech is based on the short-time Fourier transform (STFT)^34^ or Wavelet transformation^35^, which generates the magnitude and phase spectrogram. Distorting either the magnitude or the phase spectrogram would affect the intelligibility of speech. Therefore, a systematic and targeted distortion of TFS cues could be achieved by systematically distorting the phase spectrogram while maintaining the magnitude spectrogram. To further examine the neural mechanism underlying TFS processing for speech perception, we adopted the STFT approach to systematically distort the TFS cues in speech. The magnitude and phase spectrogram of natural speech was calculated with fine time resolution. The phase was shifted to various extents for sentence reconstruction. The reconstructed sentences featured systematic distortion in TFS and were used to (1) test speech perception in normal hearing listeners in both quiet and noisy listening conditions and (2) test neural activities in the auditory neurons in animals.

Our study focused on the neural mechanism of TFS processing by combining neural recordings from individual auditory neurons in guinea pigs with psychophysical measures in human subjects. The TFS cues of sentences from the Hearing in Noise Test (HINT) and the QuickSIN™ test were modified to various extents. The modified speech signals were used for speech perception tests in normal hearing listeners in both quiet and noisy conditions. These same sentences were used to evoke neural activity in well-identified auditory neurons of the inferior colliculus (ICC) in guinea pigs. Through this direct comparison between behavioral and neural data, our results bridged the perceptual role and neural coding of TFS cues in speech, allowing us to uncover the essential neural mechanism for TFS perception.

## Results

### TFS cues were distorted by phase shifting

Speech sentences with altered TFS cues were designed with little distortion of the speech E (see Methods for details). For the animal experiments, the original speech sentences (no noise presented) were selected from the HINT test and referred to as sentence type 1 (S1). A 64-channel spectrogram of each original speech sentence (S1) was calculated and the six frequency bands with the maximum energy were then selected for sentence reconstruction. Sentences reconstructed with the 6 frequency bands selected from the original S1 were referred as sentence type 2 (S2). To alter the TFS, the phase information within the six selected channels was distorted to various extents. Phase information was randomly shifted within half a period to reconstruct sentence type 3 (S3). Phase information was randomly shifted within a whole period to reconstruct sentence type 4 (S4). Phase information was also replaced by a random number to reconstruct sentence type 5 (S5). Phase information was set to zero to reconstruct sentence type 6 (S6). Finally, the magnitude of each sentence type was normalized to the magnitude of the original spectrogram.

**Figure 1:**
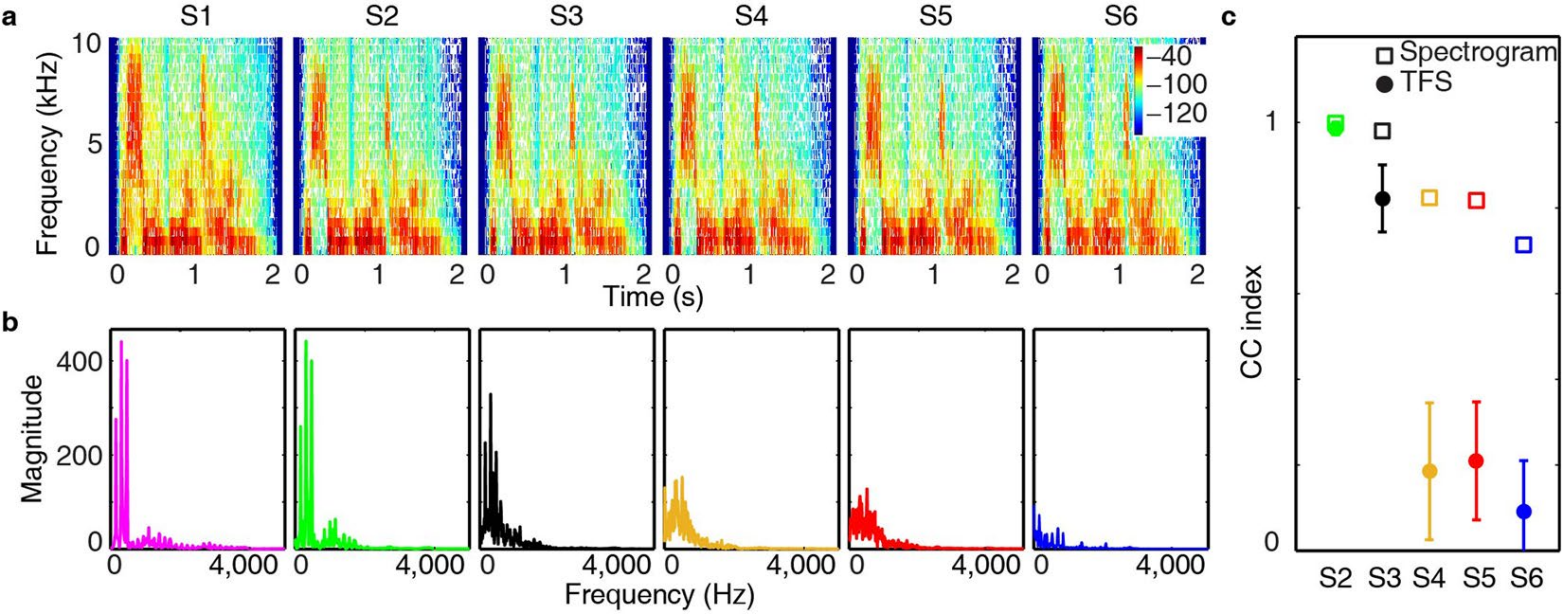
The narrow band spectrogram of S1 to S6 shows the energy of the sentence as a function of time and frequency (**a**). The CC index was calculated between the spectrogram of S1 and S2–S6 (**c**). The second “i” from the sentence “the silly boy’s hiding” was selected as an example. The spectra of “i” from 0 to 5000 Hz were plotted for each type of sentence (**b**). Similar color coding was used for all following figures. The representative peaks in S1, S2, and S3 share the same frequencies, although the magnitudes were slightly different. For each voiced syllable, the CC index was calculated between the spectra of S1 and S2–S6. The CC index was averaged across all voiced syllables (n = 9) in the sentence. The averaged CC index and the standard deviation were plotted for each sentence as solid circles and error bars (**c**). The CC index was also calculated between the spectrogram of S1 and S2–S6, as shown in open squares (**c**).

This phase distortion severely degraded the TFS, but not the E of the speech. To illustrate this point, a sentence from the HINT test was used as an example. This sentence was “the silly boy’s hiding” and it was 2 seconds long. Each sentence type, S1–S6, can be compared as a function of frequency and time via its corresponding spectrogram (Fig. 1a). Visual inspection of the spectrograms showed relatively similar magnitudes, envelopes, and spectral contents. Furthermore, the spectrograms of S2–S6 were strongly correlated with S1, with the Pearson product-moment correlation coefficient (CC) index above 0.7 (Fig. 1c). Namely, the E was well preserved from S1 to S2–S6. This was supported by the neural histograms (see Supplementary Figs S1 and S2), which showed comparable responses to all six speech signals, demonstrating that the E of S1–S6 was processed similarly in auditory neurons.

To further examine the TFS, sentences were divided into smaller time sections, for example, the second “i” from the sentence “the silly boy’s hiding”. A Fourier transformation was applied to this voiced syllable in each sentence type, S1–S6. The spectrum for “i” in sentence S1 showed three large representative peaks in the frequency range from 50 to 500 Hz, i.e. the *F*_0_, the 1^st^ and 2^nd^ harmonic of *F*_0_. These TFS cues seen in the S1 spectrum were distorted to different degrees in S2–S6, particularly affecting *F*_0_, the 1^st^ and 2^nd^ harmonic of *F*_0_, i.e. the periodic part of TFS of this syllable (Fig. 1b). This dominating temporal periodic pattern was generally preserved in S2, i.e. the representative frequency peaks were observed in the S2 spectra with similar magnitudes. In other words, sentences reconstrued with fewer frequency bands did not degrade the periodic cues of TFS. This periodic pattern was also seen in S3, though the magnitudes of the peaks were slightly decreased. This indicated that phase shifting within half a period didn’t eliminate the periodic cues of TFS. The TFS patterns were further diminished in S4, S5, and S6. Apart from these representative frequency peaks, the spectrograms of S1 to S5 were well matched in a contiguous spectral regions^4^. The CC index was calculated between the original and altered speech signals for each of the selected voiced syllables from the speech sentences. For example, there were nine selected voiced syllables “th, e, i, lly, b, oy, i, d, ing” in the sentence “the silly boy’s hiding”. The averaged CC index across the nine syllables was calculated, as well as the standard deviation (Fig. 1c). S2 and S3 were strongly correlated with S1, with an average CC index above 0.7. However, S4, S5, and S6 showed weak correlation to the original speech, with an average CC index around 0.2.

### Speech perception was affected by TFS in quiet and noise

**Figure 2:**
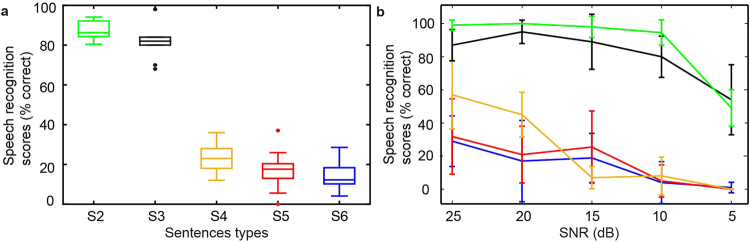
Speech recognition was tested in normal hearing subjects with five types of reconstructed sentences, S2 to S6. The scores for all test subjects for speech in quiet were shown in the box-and-whisker plot (**a**). The maximum, median, minimum, first and third quartiles were also shown in the plot. Dots outside whisker boxes were outliers not included in according boxes, but still considered in comparison. Speech recognition scores were also obtained for speech in noise for five different SNRs: average scores and standard deviations are plotted for all subjects (**b**). Five types of speech files were presented in a random order for each condition to alleviate possible training effects or tiredness of the subjects from the results.

Speech intelligibility of the altered sentences was examined in normal hearing subjects using sentence lists from the QuickSIN™ test, both in quiet listening environments and with background noise. The original sentence, S1, was not tested since subjects were normal hearing listeners. The results of the speech test for S2–S6 in quiet were plotted as a box plot, as shown in Fig. 2a. Speech intelligibility was near perfect for S2 (with reduced number of frequency bands). Speech intelligibility was over 80% on average for S3 (phase shifted within half a period). However, speech sentences became hardly intelligible for S4 (phase shifted within a full period), S5 (phase replaced by a random number), and S6 (phase replaced by 0). The average scores for correct answers and the corresponding standard deviations were 87% ± 4% for S2, 81% ± 8% for S3, 23% ± 7% for S4, 17% ± 10% for S5, and 14% ± 8% for S6. Speech performance decreased systematically with TFS distortion. Differences in outcomes were evaluated between neighboring sentences using Wilcoxon signed rank test with 95% confidence interval. The p-value at 9 degree of freedom is taken as the standard for statistical analysis. A p-value smaller than 0.05 was considered statistically different between conditions. The p-values were 0.061 between S2 and S3, 0.002 between S3 and S4, 0.049 between S4 and S5, 0.193 between S5 and S6. The significant difference between speech score for S3 and S4 suggested that speech perception in the present experiments was affected by the distortion in the TFS.

Speech recognition was also tested with sentences from the QuickSIN™ test for noisy listening conditions with five different signal-to-noise ratios (SNRs), ranging from 5 dB to 25 dB in 5 dB steps (details seen in Methods). The averaged speech recognition scores and standard deviations were calculated (Fig. 2b) for five speech sentences (S2–S6). Speech performance decreased with the reduction of SNR for all tested sentence types. The speech recognition scores also clustered into two groups: S2 and S3, as one group with overall high scores for all SNRs, and S4 to S6, as another group with poor speech scores. The separation of the two groups clearly showed that speech recognition in noisy listening environments was superior when the TFS was preserved: S2 and S3 were better recognized than S4, S5, and S6. With an SNR of 5 and 10 dB, the speech signal was almost unintelligible for S4, S5, and S6.

### Neural phase locking to TFS

**Figure 3:**
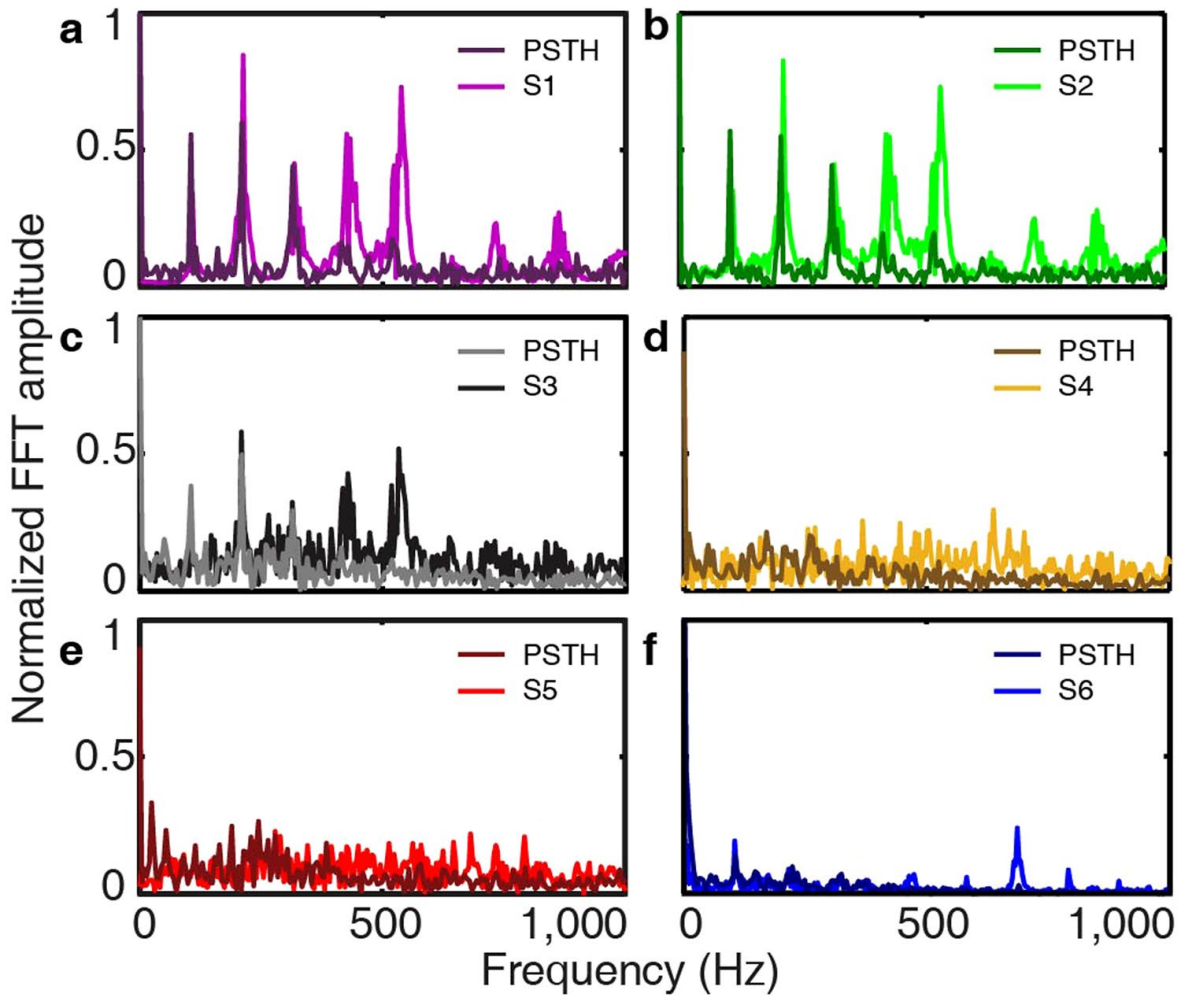
Spectra of the acoustic stimuli were plotted along with the spectra of their corresponding PSTHs calculated from the neural activity obtained during acoustic stimulation from one representative unit in the guinea pig ICC. The best frequency (BF) of the unit was 600 Hz. A voiced syllable “o” from “the silly boy is hiding” was selected (0.714 to 0.914 s post the onset of each sentence). S1 to S6 were shown in a to f. The magnitude of spectra was normalized to the maximal magnitude with 1000 Hz to allow a direct comparison. The first three dominating peaks in S1 (a), S2 (b) and S3 (c) were at 105, 210, 315 Hz respectively.

The coding of TFS cues in the speech signal was also examined at the level of individual auditory neurons. The neural activity in response to all six types of sentences was recorded from well-defined single units in the ICC of guinea pigs. Neural coding of the period cues of TFS was examined by neural phase locking^5^. The phase locking patterns to the TFS cues could be derived from the spectral analysis of the post-stimulus time histogram (PSTH) (for details see Methods). A voiced syllable “o” from “the silly boy is hiding” was selected: time section from 0.714 to 0.914 s post the onset of each sentence and PSTH. A Fourier transformation was applied to the neural PSTHs to extract the phase locking pattern. The spectra of the PSTHs evoked by S1 and S2 (Fig. 3a,b) contain three large peaks at 105, 210, 315 Hz respectively. These large peaks indicated that the neural discharge was phase locked to these frequencies. These frequencies corresponded to the frequencies seen in the FFT of the acoustic signal: the *F*_0_, as well as the 1^st^ and 2^nd^ harmonic of *F*_0_. Neural PSTHs showed identifiable peaks at 420 and 525 Hz with relatively smaller magnitude when compared with the first three peaks in the spectra of S1 and S2. The PSTH evoked by S3 also contained peaks at 105, 210, 315 Hz, but not at 420 and 525 Hz (Fig. 3c). Despite selecting only six frequency bands (S2) and shifting the phase within half a period (S3), the speech signals still showed the dominant frequency peaks seen in the original sentence (S1). Thus, the frequencies within the TFS cues were well preserved from S1 to S2 and S3, yielding phase locking in the auditory neurons, as well as normal speech recognition in humans (Fig. 2).

On the contrary, in response to S4, S5, and S6, the spectra of neural PSTH did not show any large representative frequency. Namely, no robust phase locking was seen (Fig. 3d to f), which was likely due to the absence of the periodic cues in the TFS resulting from altering the phase within a whole period (S4), or more (S5 and S6). The disruption of the periodic pattern in the TFS resulted in the absence of neural phase locking and speech recognition in humans (Fig. 2). In other words, the periodic pattern in the TFS of speech was encoded by the auditory neurons through phase locking.

**Figure 4:**
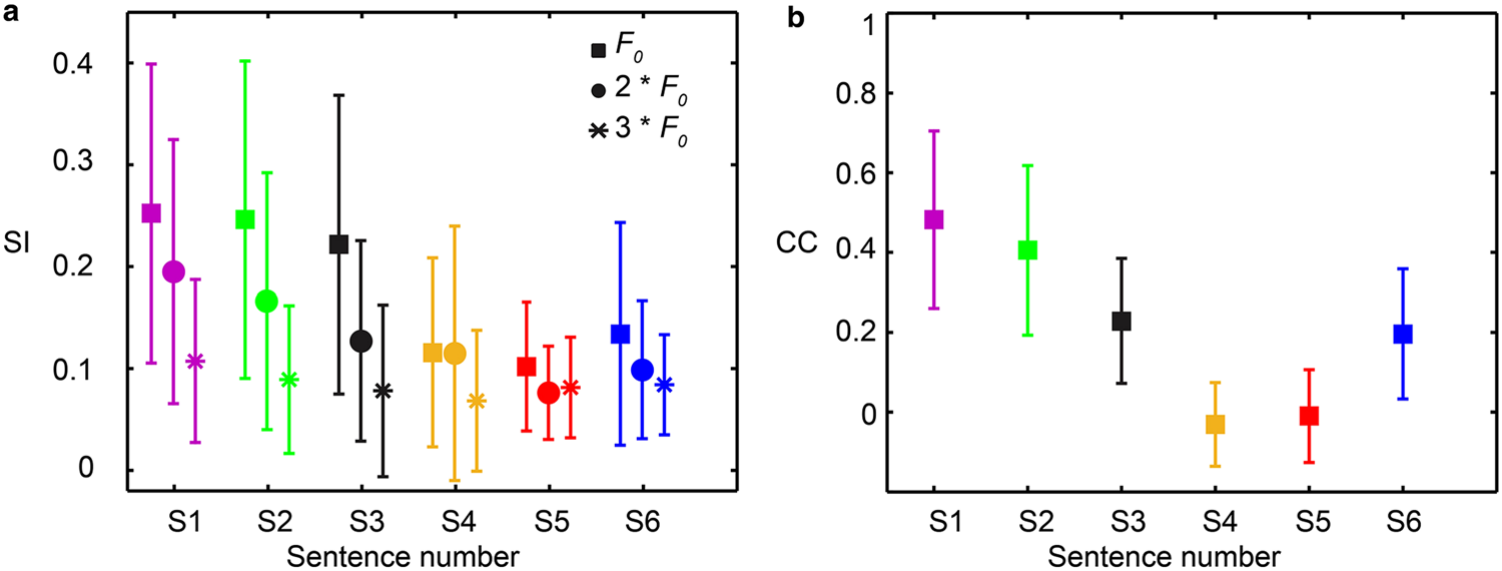
The average and standard deviation of the SI of 36 single units is shown for *F*_0_ and the 1^st^ and 2^nd^ harmonic of the selected voiced syllable “o” from “the silly boy is hiding” (**a**). The average and standard deviation of the CC between the spectra of neural PSTH from 36 single units and the corresponding acoustic spectra (**b**). The BF of these neurons ranged from 600 Hz to 3600 Hz.

Similar results have been observed in response to other voiced syllables, as well as in other ICC neurons with different BFs. Phase locking was quantified by the synchronization index (SI), i.e., the ratio between the magnitude of the frequency that neurons phase locked to and the total number of discharges recorded^30,36,37^. For each sentence type, the SI was calculated at the first three frequencies of the given syllable. The SI was averaged over 36 individual neurons (Fig. 4a). SI values at the *F*_0_ were comparable for S1, S2, and S3, but dropped significantly for S4, S5, and S6, indicating a loss of phase locking. Differences in outcomes were tested with the Student’s t-test and were statistically significant for S3 and S4 at the *F*_0_ (p = 0.0005). The CC between the spectra of PSTHs and speech signals was also calculated for all 36 single units (Fig. 4b). A systematic decrease in average CC was shown from S1 to S5. S6 showed a relatively large CC, which might be because the *F*_0_ was still visible, although the magnitude was largely decreased compared to the original sentence, S1 (Fig. 3f). This small *F*_0_ induced a small phase locking response, resulting in a large CC, as well as a relatively large variation in the SI values at this frequency (Fig. 4a).

## Discussion

The results support the current view that phase locking is indeed an important factor to process TFS information. The results obtained from human testing have demonstrated that speech intelligibility in quiet could be deteriorated when the phase shifting range was a whole period, but could be maintained when the phase shifting was within half a period. Phase shifting within half a period also showed better understanding in noise. Corresponding experiments in guinea pigs have shown that individual auditory neurons exhibit phase locking patterns to the TFS in speech that disappear with the degradation of TFS in reconstructed sounds. Overall, speech remains intelligible for a phase shift less than half a period (S3). Previous studies have shown that phase distortion compromised speech perception^34^. The results from this study demonstrated that some changes in TFS are tolerated before performance changes drastically. The maximum tolerable shift in phase was within half a period. In terms of acoustic properties, phase shifting within half a period maintained the periodic pattern of speech TFS. The preserved TFS also evoked phase locking in auditory neurons. This observation is consistent with the findings of neural phase locking. Auditory neurons tend to fire within a certain range relative to one sinusoidal cycle, not just to a single fixed time point of each cycle. The range is a quarter to half of a sinusoidal cycle^38^. Such localized firing within a time range could be beneficial for pitch detection of auditory neurons^39,40^. It also indicates that a fixed rate of stimulation is not necessary when trying to include TFS in cochlear implant coding strategies. The results show, from both acoustic and neural processing perspectives, that a certain range of phase distortion, i.e. within half a period, does not affect speech intelligibility.

Sentences with TFS distortion were played to humans and guinea pigs for parallel comparison. Previous studies have demonstrated that the auditory tuning curves obtained from action potentials were comparable between humans and guinea pigs. Specifically, these studies showed that there is a general broadening trend from high to low frequencies^41–43^. The sharpness of tuning measured from action potentials was reported to be smaller than those obtained from single-fiber tuning curves in guinea pigs^44^ and psychoacoustically in humans^45^. On the other hand, new otoacoustic measurements indicated that cochlea tuning in humans is sharper than other mammals, including guinea pigs^46^. To overcome the controversial differences of auditory tuning between humans and guinea pigs, the stimulation level was high to evoke broad tuning for both species. The sound level used for human speech perception tests was 61 dB (re 20 µPa). The sound level used for neural activity recording was 80 dB (re 20 µPa). Thus, the auditory systems of humans and guinea pigs share similar broad width of tuning at these sound levels.

The spectrogram of S1 to S6 was comparable regarding magnitudes, envelopes, as well as relative spectral contents as demonstrated in Fig. 1. However, there was a significant difference between the speech perception evoked by these sentences. The difference in speech intelligibility was consistent, with the distortion in periodic patterns of TFS. Previous studies have demonstrated that with only temporal cues of 4 frequency bands, speech was intelligible for normal hearing listener^4^. These temporal cues were extracted by a low-pass filter with cutoff frequencies at 16, 50, 160 or 500 Hz. The slope of the low-pass filter was 6 dB per octave. Thus, the temporal cues may contain energy of 50 to 500 Hz, i.e. the periodic cues of TFS including the *F*_0_, as well as the 1^st^ and 2^nd^ harmonic. Some other studies have shown that speech with only the E (<50 Hz) of 16 frequency bands was able to maintain intelligibility for normal hearing listeners in noisy condition^3,47,48^. Although TFS may not be seen within each frequency bands, the large number of frequency bands may have compensated for the temporal loss through the place coding. However, our study showed that speech signals with periodic cues of only six channels out of 64 frequency bands could maintain intelligibility in noise. The small number of frequency bands was selected to mimic the n-of-m coding strategy for cochlear implants. The number of independent frequency bands for cochlear implant users is usually 4 to 8. Thus, cochlear implant users may benefit from a coding strategy containing periodic patterns of TFS in addition to the E.

Although not examined systematically, most test subjects (6 out of 10) could recognize words with strong consonantal syllables, such as “sh” and “s” from audio files, with large phase distortion under low or medium noise levels (>15 dB SNR), but hardly recognized vowels. This might be because neural phase locking is evoked mainly by the periodic pattern in vowels, but not for high frequency consonants. Thus, the periodicity distortion had smaller effects on consonantal syllables than on vowels. The zero phase condition (S6) gave a robot-like sound with less tonal information according to the subjects, which decreased the scores for speech recognition (Fig. 2). This is consistent with previous findings that temporal periodicity is important for tonal and vocal information in audio signals^2^.

During the test, some subjects (4 out of 10) stated that they could not tell the gender of the speaker for S4, S5, and S6 (phase shifted within one period or more). Typically, the female voice was identified as a male. This is consistent with previous studies showing that adding TFS cues could increase gender discrimination with limited frequency bands of signal processing^49^. Since gender discrimination is mainly determined by the *F*_0_ perception^50,51^, having *F*_0_ preserved in the speech might benefit gender discrimination. During the experiment, the test subjects reported that they grew accustomed with the speech sentences. This might result in an increase in speech intelligibility. The speech intelligibility tests were conducted firstly in the quiet environment, and then in noise (five levels of SNR). Thus, across the two types of speech tests (in quiet and in noise), the training effects might have caused the score to increase for S4 in noise at 25 dB SNR. On the other hand, within the two types of speech recognition test (in quiet and in noise), we have taken three approaches to minimize possible training effects: (1) 3 sets of practice speech files were presented to the subjects prior to the experiment to familiarize the subjects with reconstructed speech. (2) For speech perception tests in quiet, the order of S2 to S6 was randomized to alleviate tiredness from sentences difficult to understand. (3) For speech perception tests in noise, both the order of S2 to S6 and the order of 5 SNR levels were randomized to alleviate learning effects. Across speech tests in quiet and noise, the learning effect was likely the reason behind the increased score for S4 in noise at 25 dB SNR. Within the speech tests in noise, the randomization of speech files minimized the learning effects so that it was not influential enough to alter the trend of the scores as SNR decreased. Additionally, with the randomization of speech files, there was a clear separation of speech scores: S2 and S3, as one group with overall high scores for all SNRs, and S4 to S6, as another group with poor speech scores.

Studies have suggested that listeners with sensorineural hearing loss may have an increase in the perceptual salience of envelope structure that could adversely affect speech perception in fluctuating background noise through loudness recruitment^52–54^. People with moderate flat hearing loss showed decreased ability to use TFS cues^3^. It has also been suggested that the temporal precision of E coding could be enhanced with hearing loss at equal stimulus sensation level in chinchillas^55^. In addition, pronounced distortions in the tonotopic coding of temporal cues in auditory nerve fibers have been found in chinchilla after noise-induced hearing loss^56^. Our study has shown that periodic cues of TFS are essential for speech perception in normal hearing listeners in both quiet and noisy condition. It would be interesting to examine the perceptual role and neural coding of periodic patterns in the TFS in subjects with different levels and types of hearing loss.

In summary, our study provides direct evidence that neural phase locking is the underlying mechanism for processing periodic patterns in the TFS, which is essential for speech recognition in quiet and noisy conditions. Shifting the phase spectrogram of STFT within half a period would partially maintain the periodic pattern of TFS in speech, yielding speech perception in normal human listeners and phase locking in guinea pig auditory neurons. Thus, preserving the partial periodic pattern of TFS could benefit speech intelligibility in noise, tonal perception and gender discrimination. Cochlear implant users may benefit from a coding strategy containing periodic patterns of TFS in addition to the E.

## Methods

### Signal processing

**Figure 5:**
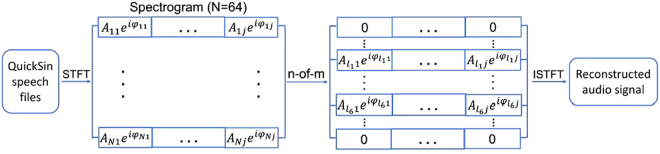
Flow-chart of signal reconstruction for TFS distortion. The input audio file was first analyzed by calculating the STFT. The resulting spectrogram contained 64 frequency bands. Applying the n-of-m method, the six frequency bands with highest energies were selected while eliminating other frequency bands to reconstruct new speech sentences. Magnitudes of selected channels were preserved, but phase values were modified under five conditions. The modified spectrogram was then used to reconstruct a new audio signal with phase distortion.

Speech sentences were processed through STFT and the phase information was manipulated to create acoustic stimuli with TFS distortion. The flow chart in Fig. 5 shows the general signal processing procedure. An STFT was applied to the speech sentence to separate speech into contiguous frequency bands using MATLAB built-in function *spectrogram()*. The parameters for the STFT were selected that the output $S(f,t)$ was an ${\rm{n}}\times {\rm{m}}$ matrix of complex numbers with 64 frequency bands (n = 64), shown in Fig. 5. The time window for STFT was around 1.5 ms, which mainly provide targeted TFS distortion below 689 Hz^34^. This frequency range was dominated by the periodic patterns of TFS.

Each element $S(f,t)$ was converted into a combination of magnitude $|S(f,t)|$ and phase $\phi$. To reconstruct speech sentences, the reverse STFT was applied. To mimic the coding strategy in cochlear implants, the number of frequency bands was reduced from 64 to 6. The frequency bands were selected using the n-of-m method: the six highest magnitudes were selected and reselected every 4 ms. All other frequency bands were eliminated. The 6 bands were then used to reconstruct speech sentences. To distort the periodic pattern, the phase values of the selected frequency bands were changed according to five conditions: 1) original phase values ${\pi }_{0}$, 2) random phase shift in a range of $[{\pi }_{0}-\frac{\pi }{2},{\pi }_{0}+\frac{\pi }{2}]$, 3) random phase shifts in a range of [*π*_0_ − *π*, *π*_0_ + *π*], 4) random numbers within [−*π*, *π*], and 5) fixed numbers, zeros. These five conditions were used to reconstructed S2 to S6. With the original magnitude in the selected channels and the distorted phase values, equation 1

1 $$S(f,t)\,=\,\,|S(f,t)|{e}^{i\phi }$$

was used to reconstruct complex numbers in the new spectrogram $S^{\prime}$. By calculating the inverse-STFT from the new spectrogram, S2 to S6 was reconstructed. The noise masker used for the speech in noise tests were 4-talker babble and was part of the track. Therefore, both the target speech and noise maskers were processed through phase distortion. For the sentences from the HINT test one channel contained the noise signal, the other channel the speech signal. For the speech in noise experiments no sentences form the HINT test were used.

### Human test subjects and approach

#### Ethics statement

The study was approved by the Northwestern Institutional Review Board (IRB), filed with the version number STU00201779. Informed consent was obtained from all participants at the time of their enrollment in the study. All experiments were performed in accordance with relevant guidelines and regulations.

#### Subjects

For the experiments, 10 self-reported normal hearing native English-speaking subjects (3 females and 7 males, average age of 28 ± 8.8) were recruited. Normal hearing was confirmed by conducting a hearing test (see also supplementary information).

#### Sentence test

The speech test was conducted in a quiet room with a noise level typically less than 40 dB (re 20 µPa). Speech audio files were played to subjects via calibrated headphones at 61 dB (re 20 µPa). A practice test with three sentences, each under a different phase distortion condition that subjects may come across in the test, were played first to the subjects so that they could familiarize themselves with the process. Next, ten new sets of sentences (two sets for each sentence types from S2 to S6) were played in a random order. Subjects were asked to repeat what they heard as accurately as possible after every sentence. Also, during the test, subjects were encouraged to describe their experience of the audio files, including pitch, timber and gender recognition.

The QuickSIN™ user manual provides a score sheet for each of the sentences, which was used for evaluation. Percentages of correct meaningful words repeated by subjects were calculated as the score for each sentence. To evaluate performance difference among different phase distortion conditions, the correctness of all SNR levels within the same phase-changing condition were summed and then compared for statistical analysis.

### Animal experiments and approach

#### Ethics statement

10 albino guinea pigs (200–800 g) of either sex were used in the experiments. Care and use of animals were carried out in accordance with the NIH Guide for the Care and Use of Laboratory Animals and were approved by the Animal Care and Use Committee of Northwestern University.

#### Surgery and electrode placement

Surgical procedures have been routinely performed in our laboratory^57–59^. Animals were anesthetized and the inferior colliculus of the guinea pigs was surgically accessed. A 16-channel thin film microelectrode (NeuroNexus, Ann Arbor, MI) was inserted for recordings into the central nucleus of the ICC perpendicular to its iso-frequency planes. A detailed description of the anesthesia and the animal monitoring is provided in the supplementary information.

#### Measurements

After a stable single unit in the ICC was identified, its BF was determined (detailed information seen in the supplementary information). Next, a sequence of 6 speech sentences was played to the left ear while the neural activity was recorded. S1 is the original speech sentence from the HINT test, “the silly boy is hiding”. S2 to S6 were reconstructed based on the signal processing method described earlier. For each identified neural unit, the speech sentence sequence was repeated at least 100 times to achieve a sufficient number of action potentials for analysis. The timing of the action potential of individual ICC single units was sorted offline and transferred as neural pulses for analysis (see also data analysis below).

#### Data analysis

Firstly, neural coding of TFS was investigated. Sentences were divided into time sections based on the spectral properties to study the phase locking in ICC neurons. For example, a 200 ms time section containing “o” from “the silly boy is hiding” was selected for all six sentences (Fig. 3). A Fourier transformation was performed on the selected speech sentence section to extract the periodic patterns of TFS (Figs 1 and 3). The phase locking patterns to the periodic patterns of TFS could be derived from the spectral analysis of the neural PSTH. A Fourier transformation of the PSTH was evaluated. If dominating peaks were observed in the spectra (Fig. 3a,c,d), the positions of these frequency peaks were taken to represent the frequencies at which neurons showed phase locking. SI was calculated to examine phase locking strength for each frequency of interests. A CC index could be calculated between the spectra of the acoustic signal and neural PSTHs. If no dominating peaks could be observed, phase locking was considered as absent.

## Electronic supplementary material

Supplementary Information
