# Supplementary material for: Distorting temporal fine structure by phase shifting and its effects on speech intelligibility and neural phase locking
Source: Sci Rep. 2017 Oct 17;7:13387. doi: 10.1038/s41598-017-12975-3 (PMC5645416; doi:10.1038/s41598-017-12975-3)
Supplement: Supplementary file 1 — Supplementary Information [file 41598_2017_12975_MOESM1_ESM.pdf]

Supplementary Information

Distorting temporal fine structure by phase shifting and its effects on speech intelligibility and neural phase locking

Yingyue Xu[a*], Maxin Chen[b*], Petrina LaFaire[a], Xiaodong Tan[a], Claus-Peter Richter[a-c]

Hearing test for human experiments

Hearing evaluation

To evaluate the hearing performance of subjects, their pure-tone hearing thresholds were determined, using an Audiometer GUI built in MATLAB. A sequence of two seconds pure tones at 250, 500, 800, 1000, 2000, 4000, 8000, and 10000Hz was played to the test subjects. After a pure tone of each frequency was played, subjects were asked to respond whether they've heard the sound or not. The level of the tone was varied until the test person just identified the tone. Results were compared to a standard reference group verifying that no hearing loss exists. Subjects were tested with their left ear first and then right ear. Sounds played to both ears were the same in settings.

Devices and software used in this study were: Macbook Pro; MATLAB R2014b; headphone (Beyerdynamic DT990); sound level meter (Radio Shack) for course calibration and the Bruel& Kjaer 1/8 inch microphone for off-line recalibration of the headphones.

Animal experiments and approach

Surgery and electrode placement

All animals were anesthetized during experimental procedures. Anesthesia was induced by an intraperitoneal injection (i.p.) of urethane (0.9 mg/kg) in a 20 % Ringer's Lactate (RL) solution. During the experiment, the level of anesthesia was assessed by a paw withdraw reflex at 15 mins intervals. Supplemental doses of ketamine (40 mg/kg) and xylazine (2.5 mg/kg) were given with RL solution when needed. The body temperature of the animals was maintained at 38°C with a heating pad. A BM3-Vet system (Bionet Co. Ltd, Seoul, Korea) served to continuously monitor the animal's vital signs, including heart and respiratory rates and blood oxygen saturation.
The frontal bony skull of the animal was surgically exposed and mounted to a stereotactic head holder (Stoelting, Kiel, WI) using dental acrylic (Methyl methacrylate, Co-oral-ite Dental MFG Co., CA). The inferior colliculus was surgically accessed through an opening on the right parietal bone just dorsal to the temporoparietal suture and just rostral to the tentorium. A 16-channel electrode (A1x16-5mm-100-177, NeuroNexus Technologies, Ann Arbor, MI) was used for single neuron recordings in the central nucleus of the inferior colliculus (ICC). The electrode was advanced into the ICC at 45° off the parasagittal plane using a 3D micromanipulator (Stoelting, Kiel, WI). After the initial placement, acoustic clicks at various levels were presented to the left ear. Neural activities were monitored on an oscilloscope to confirm whether the recording is from individual ICC neurons. The electrode was further advanced into the ICC in steps of

400 µm until a single neural unit was identified. The position of the electrode was maintained for further measurement.

Characteristic frequency (CF) assessments

After a single neuron had been identified, its CF was roughly estimated using sweep tones. Then pure tone ranging from 2 octaves above and below the estimated CF was presented to accurately assess the CF. The pure tone was given at levels ranging from 0 to 80 dB attention from the maximum speaker output (110 dB SPL) with 5 dB step. The number of evoked action potential was calculated to assess the CF.

Envelope coding remain comparable after phase shifting

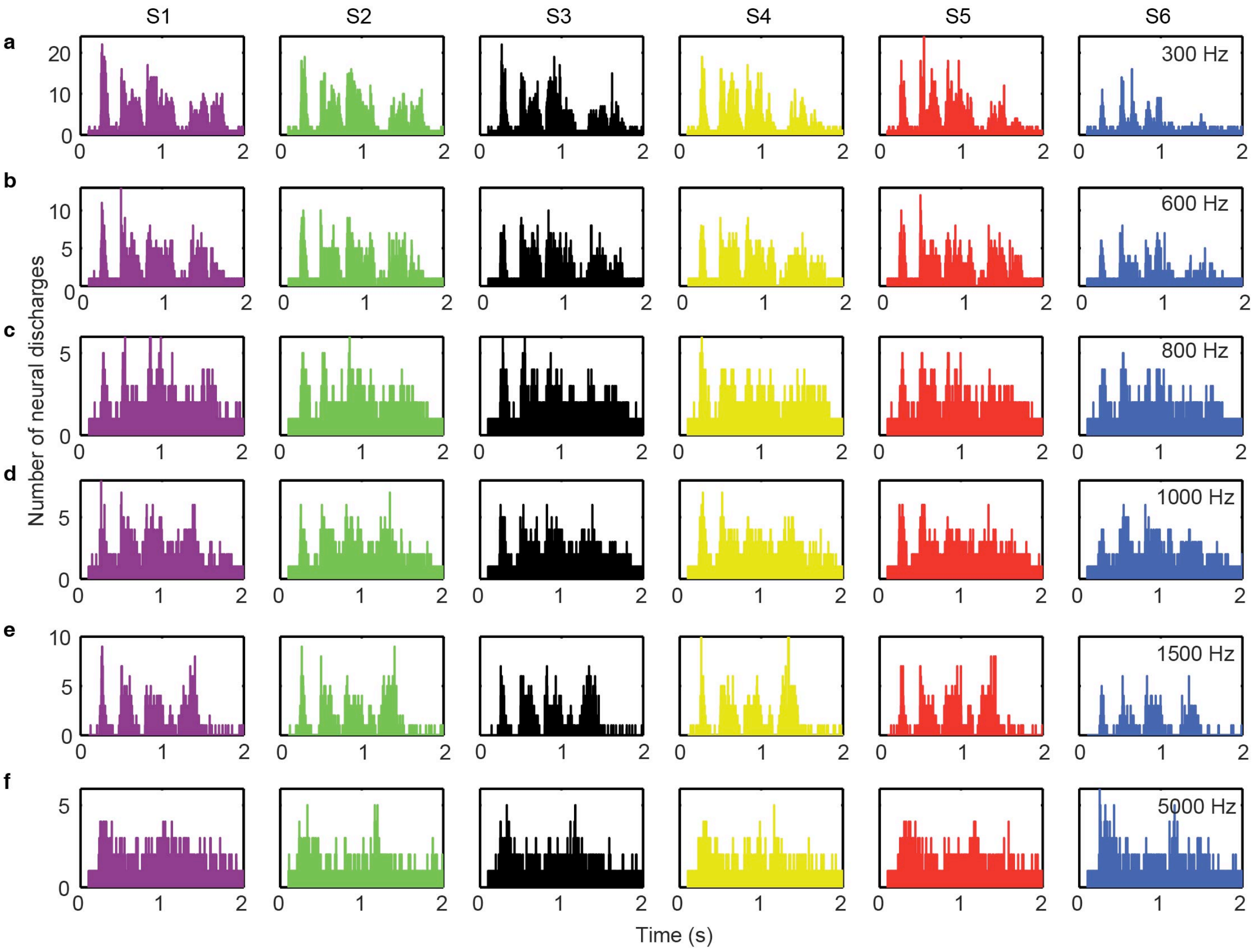

Fig. S1. PSTHs constructed from the neural activity recorded in the ICC while the sentences S1 to S6 were played to the ear. Rows a-f show the results for six representative ICC neurons with different BFs.

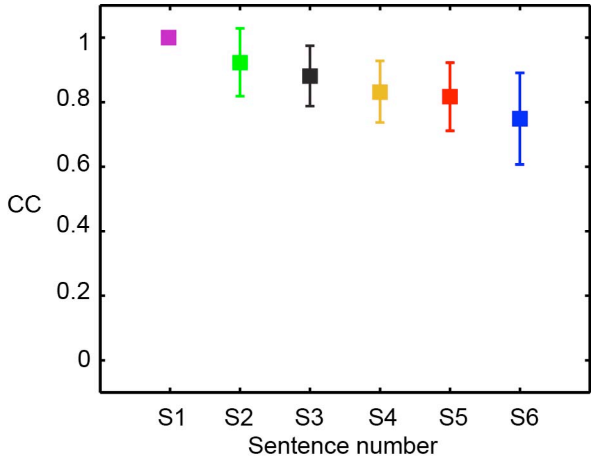

Fig. S2. The similarity of the neural PSTHs for S1 to S6 was compared using CC index across all individual neurons. The average CC index between neural PSTHs evoked by S1 and S1 to S6 was plottet with its standard deviation. The time window for PSTH was 10 ms.

The effects of the n-of-m strategy and phase manipulation on envelope, periodicity, and TFS within frequency bands:

To set the ground for discussion, a comparison of unfiltered acoustic signal and the filtered acoustic signal is shown in Fig. S3 to show the frequency range from 50 to 500 Hz. The phase distortion approach mainly targets the frequency range from 50 to 500 Hz, i.e., the periodicity defined in Rosen's 1992 paper. This terminology is also used by other researchers (Rosen,1992; Langner, 1992; Green et al., 2005; Steinmetzger & Rosen, 2015; 2017a; 2017b). Since this range is most evident in low frequency bands with vowels, the voiced syllable "o" from "the silly boy is hiding" and a low frequency band (centered frequency of 689 Hz) was picked for demonstration.

The speech signal was filtered into 16 frequency bands up to 10 kHz. The band-pass filters were relatively broad resulting from the sound level used in the experiments. The sound level used for human speech perception tests was 61 dB (re 20 μPa). The sound level used for neural activity recordings was 80 dB (re 20 μPa). These levels were relatively high, leading to a broad tuning for both human and guinea pigs. Previous studies that compared the sharpness of tuning between humans and guinea pigs reported inconclusive results. The broad tuning evoked in our study was aimed to overcome the possible difference of auditory tuning between the two species as addressed in the discussion of the manuscript.

The amplitude of the unfiltered signal and the filtered signal was plotted in its time waveform (Fig. S3 a, d). The spectral analysis of this time waveform showed the amplitude fluctuation at different frequencies (Fig. S3 b, e). The same neural response was plotted twice for the purpose of demonstration (Fig. S3 c, f). The PSTH was constructed using a 0.025 ms time window, which is short enough to capture fluctuation below 20,000 Hz based on the Nyquist–Shannon sampling theorem. Spectral analysis of the PSTH demonstrated the phase locking patterns (Fig. S3 c, f).

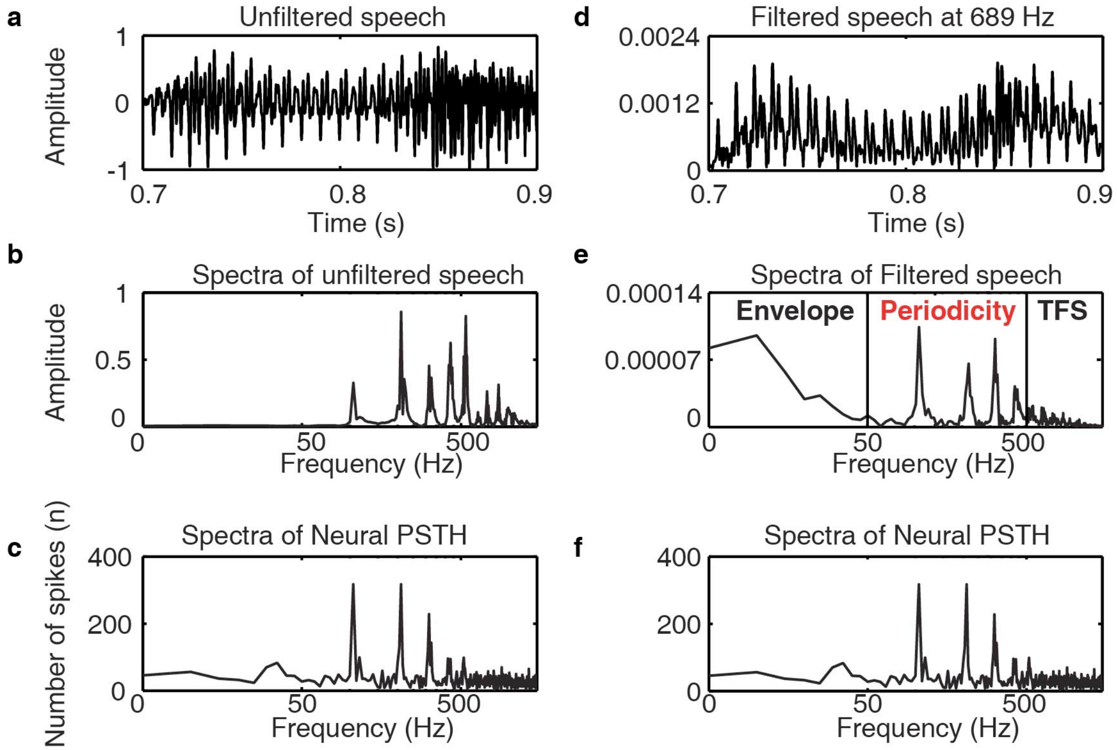

Figure S3. A voiced syllable “o” from “the silly boy is hiding” was selected (0.714 to 0.914 s post the onset of each sentence). a and d showed the time waveform of unfiltered speech and band-pass filtered speech at the center frequencies of 689 Hz. b and f showed the spectra of the unfiltered speech and band-pass filtered speech. The range of envelope, periodicity, and part of the TFS were listed. c and g showed the same spectra of a neural PSTH. The “periodicity” was referred as the periodic cues of TFS in the manuscript. The best frequency of the neuron was 600. The number of repetitions was more than 100 for the sentence.

As shown in Fig. S3 e, there are three components based on frequency ranges according to Rosen’s paper (1992):

1. Envelope: The low frequency fluctuation <50 Hz. The envelope was better observed in the band-pass filtered signal. This is because the band-passing filtering at 689 Hz emphasized low frequency components more compared to the unfiltered signal.
2. Periodicity: The 50 to 500 Hz fluctuation, which is the target frequency range of our study. Within the range of 50 to 500 Hz, there were dominating frequency peaks in both unfiltered and filtered signals, i.e. the fundamental frequency and its harmonics. Due to the recognition of recent literatures refer TFS simply as the high frequency components of auditory filter outputs. The term “periodic cues of TFS” was used to substitute periodicity in the manuscript.
3. TFS: The high frequency range from 600 to 10,000 Hz, but was not fully plotted.

The effects of n-of-m strategy and phase distortion on the output of auditory filter at the center frequency of 689 Hz (Fig. S4) was presented. For the purpose of clarification, we use "envelope", "periodicity", and "TFS" as shown in Fig. S3.

The analysis of band-pass filtering on S1 to S6 was presented in Fig. S4. The amplitude of one frequency band (center frequency 689 Hz) was plotted in its time waveform for S1 to S6 (Fig. S4 a - f). The spectral analysis of the time waveform showed the frequency component of the amplitude fluctuation for S1 to S6 (Fig. S4 g - i). The spectral analysis of neural PSTH in response to S1 to S6 was shown (Fig. S4 m - r). As demonstrated by the spectral analysis, the envelope was nicely preserved from S1 to S2, S3, S4, but not in S5 and S6. The periodicity was preserved from S1 to S2, S3, but not in S4, S5, S6. TFS was slightly altered from S1 to S2-S6, but there was no obvious pattern. On the other hand, normal speech perception was only in S1, S2, S3. Phase locking to fundamental frequency and its harmonics was only observed in response to S1, S2, S3. Thus, the speech perception results and phase locking patterns were greatly correlated with the change in periodicity, not with envelope. In other worlds, our approach has isolated the effects of the targeted frequency range 50 to 500 Hz.

The discontinuities in the signal introduced by the n-of-m strategy could be seen through the comparison between S1 and S2 (Fig. S4 a, b, g, h). The time waveform of original auditory filter output was shown in Fig. S4 a. Discontinuities in the signal introduced by the m-of-n strategy resulted a time waveform shown in Fig. S4 b. A better comparison can be seen in the spectral analysis (Fig. S4 g and h). The envelope (<50 Hz) and periodicity (50 to 500 Hz) were very comparable between S1 and S2. The TFS range was not changed dramatically. More importantly, the speech perception results for S1 and S2 are very similar. Thus, the slight alternation in TFS (> 500 Hz) did not affect the speech perception results. Of note, the neural response and the phase locking patterns evoked by S1 and S2 were similar (Fig. S4 m and n).

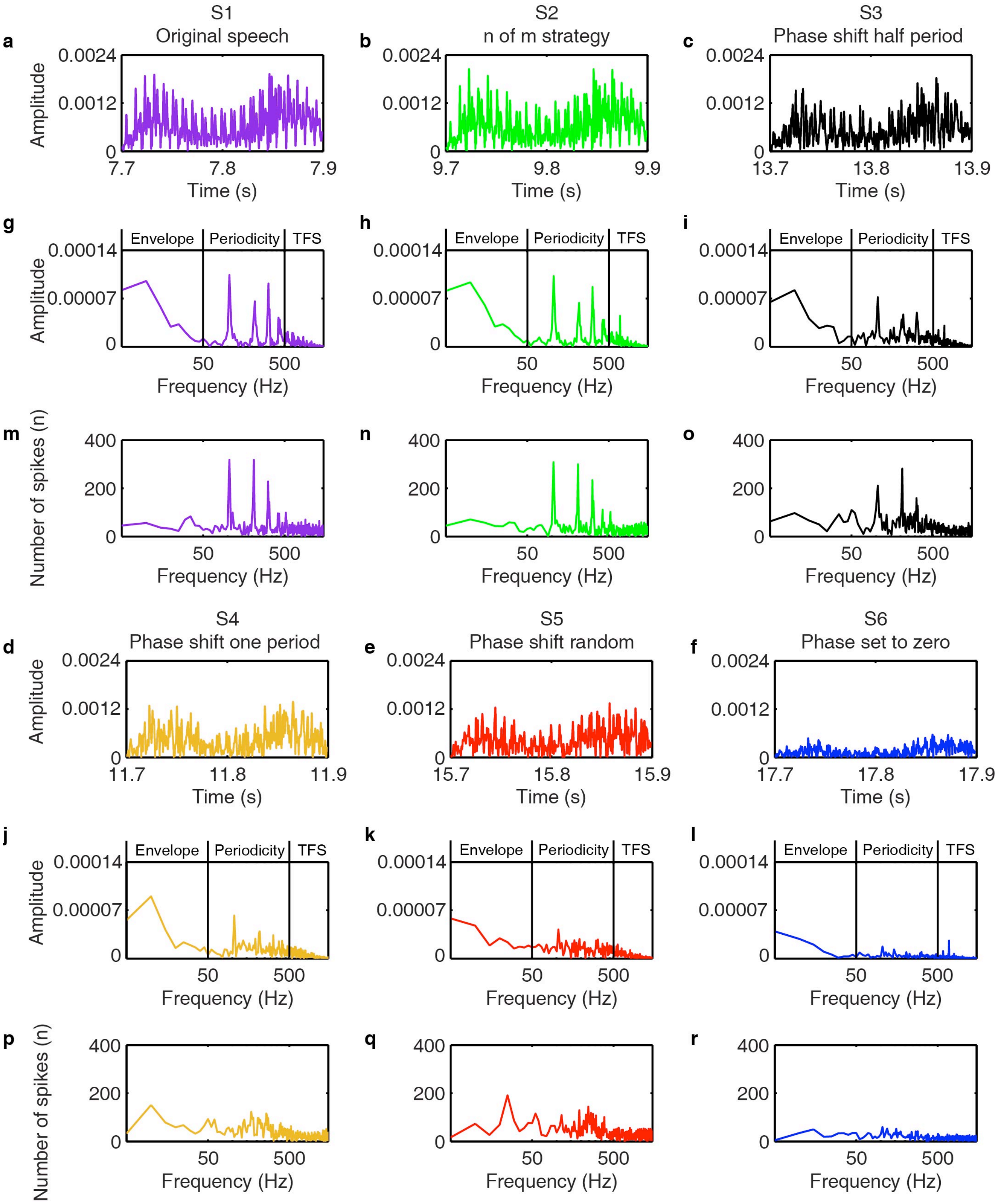

Figure S4. A voiced syllable "o" from "the silly boy is hiding" was selected (0.714 to 0.914 s post the onset of each sentence) and filtered into 16 frequency bands up to 10 kHz. One frequency band was selected as an example. Panels a to f show the time waveform of filtered S1 to S6. Panels g to l show the spectra of the filtered signal. Panels m to r show the spectra of the

PSTHs constructed from the neural responses of one neuron. PSTH was recorded with 100 repartitions of the acoustic signal.
